# Convergent and Distinct Effects of Multisensory Combination on Statistical Learning Using a Computer Glove

**DOI:** 10.3389/fpsyg.2020.599125

**Published:** 2021-01-13

**Authors:** Christopher R. Madan, Anthony Singhal

**Affiliations:** ^1^School of Psychology, University of Nottingham, Nottingham, United Kingdom; ^2^Department of Psychology, University of Alberta, Edmonton, AB, Canada; ^3^Neuroscience and Mental Health Institute, University of Alberta, Edmonton, AB, Canada

**Keywords:** sequence learning, multimodal, implicit knowledge, finger tapping, computer glove

## Abstract

Learning to play a musical instrument involves mapping visual + auditory cues to motor movements and anticipating transitions. Inspired by the serial reaction time task and artificial grammar learning, we investigated explicit and implicit knowledge of statistical learning in a sensorimotor task. Using a between-subjects design with four groups, one group of participants were provided with visual cues and followed along by tapping the corresponding fingertip to their thumb, while using a computer glove. Another group additionally received accompanying auditory tones; the final two groups received sensory (visual or visual + auditory) cues but did not provide a motor response—all together following a 2 × 2 design. Implicit knowledge was measured by response time, whereas explicit knowledge was assessed using probe tests. Findings indicate that explicit knowledge was best with only the single modality, but implicit knowledge was best when all three modalities were involved.

## Introduction

Much of human behavior relies on the ability to make predictions based on integrating multisensory input to support multi-dimensional actions and decisions. This is a key component for the control of hand motor commands, such as reaching, grasping, and object manipulation. For example, first learning to play a musical instrument involves several distinct components, such as the mapping of visual or auditory cues to motor movements and being able to anticipate the transition to the next motor movement. Initial experiences involve following along with a predetermined sequence of visual and auditory cues. Later on, this process can be planned from rehearsal or creatively reflexive. More generally, many everyday behaviors can be examined as motor command sequences that transition through a broad statistical structure (see Baldwin et al., 2008).

Several standard experimental paradigms are related to this type of learning, such as the serial reaction time task (SRTT) and artificial grammar learning (AGL). Briefly, the SRTT involves making repeated button presses following visual instructions, where the sequences are either fixed or random (Nissen and Bullemer, 1987; Karni et al., 1995, 1998; Robertson, 2007; Clark and Ivry, 2010; DeCoster and O’Mally, 2011; Schwarb and Schumacher, 2012; Stefanescu et al., 2013). In contrast, AGL involves learning implicit rules of a probabilistic transition structure, based on a finite state machine, and is used as a model of language acquisition (Reber, 1967; Perruchet and Pacteau, 1990; Reber et al., 1996; Pothos, 2007; Erickson et al., 2016), though similar procedures have also been used to study memory for sequences (Reed and Johnson, 1994; Jones and Pashler, 2007; Bornstein and Daw, 2012; Schuck et al., 2012; Altmann, 2016). Both of these paradigms, however, miss an important component relative to the skill acquisition involved in real-world behaviors: Conventional SRTT only uses fixed or random sequences, but does not have implicit rules; artificial grammar has implicit rules but does not involve sequences of motor commands. Moreover, it remains an open and important question as to whether statistical learning operates via separate modality-specific mechanisms compared to a single high-level integrated system that is multi-modal in nature (Mitchel et al., 2014).

While some prior studies have sought to integrate both SRTT and AGL procedures (Hunt and Aslin, 2001), we were particularly interested in the influence of multisensory cues and the role of motor commands in implicit and explicit statistical learning tasks. Our design was based on the idea that a general central system would need to optimally integrate visual and auditory information for both implicit and explicit components of a statistical motor-learning paradigm, whereas a set of modality-specific systems might vary in their influences on motor learning.

**FIGURE 1:**
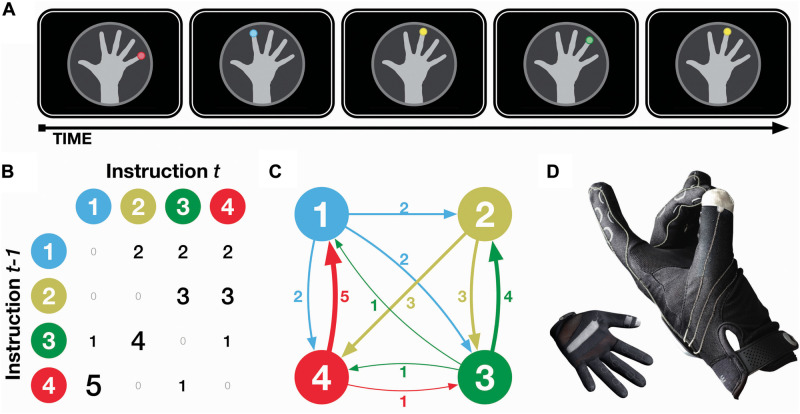
Experimental procedure. **(A)** Illustration of task design, **(B,C)** matrix and finite state schematics, and **(D)** photos of computer glove input device. Transition values and line weights correspond to the proportion (out of every six occurrences), that finger tap instruction *t - 1* will be followed by instruction *t*. Note that finger taps were never sequentially repeated (i.e., *t - 1* and *t* are never the same), nor are any transitions deterministic (i.e., no 6 s in the matrix). All fingertips occurred equally often overall (i.e., all marginals are 6/6).

Participants were presented with visual instructions to tap a specified fingertip with their thumb, with sequences of finger taps designed to follow a probabilistic transition structure, as shown in Figure 1. Transitions were designed such that some transitions were more likely, e.g., ring finger is most likely to be followed by index finger, but that all finger taps occurred equally often. Reaction time was measured using a computer glove that detected when finger taps occurred before advancing to the next instruction, allowing us to measure *implicit knowledge* of the probabilistic transitions as the experiment progressed. Participants were periodically also asked to predict the next finger tap, providing a measure of *explicit knowledge* of the probabilistic transitions. A second group of participants received both visual and auditory instructions, where a pure tone additionally accompanied the visual instruction. This comparison group allowed us to examine how additional sensory information can help or hinder learning.

A third and fourth group of participants were not permitted to make motor movements based on the instructions, and were instead explicitly asked to only observe the instructions while keeping their hands flat on the table in front of them. The timing of the instructions for these participants was yoked to participants in the prior two groups, who did make motor movements based on the finger tap instructions. These participants, however, were still probed for their knowledge of the probabilistic transitions, allowing us to examine the contribution of motor movements, i.e., enactment, to explicit knowledge of the transition structure.

In summary, by varying the learning cues presented to each group of participants, we will compare how multimodal sensorimotor information may enhance or impair learning of probabilistic sequences, in comparison to the idea of modality specificity, where unimodal information is sufficient. Furthermore, here we included both implicit and explicit tests of task knowledge, allowing for the measurement of potential of trade-offs between learning systems.

## Materials and Methods

### Participants

A total of 90 young adults (64 female; aged 18–35) participated for a $10 (Canadian) honorarium. Participants were recruited using ads posted around the University of Alberta campus. All participants were right handed (laterality quotient: *M* = 89.4, *SD* = 9.1), measured using the Edinburgh Handedness Inventory (Oldfield, 1971). Informed written consent was obtained from all participants prior to beginning the study, which was approved by the University of Alberta Institutional Review Board.

Participants were excluded for being ambidextrous (laterality < 70; *N* = 4), having insufficient English fluency (i.e., had difficulty understanding the task instructions; *N* = 1); tapping along while being in one of the Observe groups (*N* = 3), or had particularly slow response times (> 3 *SD*; *N* = 2). A total of 80 participants were included in the reported analyses.

### Procedure

Participants were randomly assigned to one of four groups, following a 2 × 2 design. Participants either wore a glove and followed the finger taps presented on the screen (Glove, “G”), or passively observed the finger tap images with presentation times yoked to another participant (Observe, “O”). Additionally, finger tap presentation screens were either accompanied by a coinciding tone (Tone, “T”), or were silent without any auditory cues (Silent, “S”). Thus, the four groups were Glove + Tone (GT), Glove + Silent (GS), Observe + Tone (OT), and Observe + Silent (OS).

The task consisted of 16 sequences (blocks) of 145 items/trials (i.e., finger tap instructions) each, for a total of 2,320 trials. Finger tap instructions remained on the screen until the appropriate tap was made, and were immediately followed by the following trial upon the tap occurring. An example of a sequence of trials is shown in Figure 1A. Transitions between the finger tap instructions are shown in Figures 1B,C. Briefly, finger tap sequences were constructed such that (a) instructions never sequentially repeated, (b) no transitions were deterministic, and (c) all instructions occurred equally often. See the caption of Figure 1 for further details.

For the participants in a Glove groups, a Peregrine glove (Iron Will Innovations Canada Inc., Lloydminster, AB) was used to detect finger tap responses. Participants’ non-dominant (left) hand was measured so the correct glove size (small, medium, or large) could be used. The glove is designed for use with computer gaming and as such only left-handed gloves are produced by the manufacturer. The design intention is for the glove to be worn on the user’s off-hand and replace a computer keyboard, with their dominant hand uses a computer mouse. However, in the current study the dominant hand was not involved in the experimental task.

A short practice task preceded the experiment to test that participants were able to successfully make finger taps that registered on the computer. In the main task, finger tap instructions remained on the computer screen until the response was made. For participants in the Observe groups, instruction presentation times were yoked to a unique participant in the corresponding Glove group, to match for presentation durations diminishing over the course of the experiment as the glove participants learned the transition probabilities. Participants were asked to keep their hands flat on the table and explicitly instructed to not make movements based on the finger tap instructions and to only imagine the movements.

Every 30–35 trials, participants were prompted to predict which finger tap instruction would occur next. Participants were shown a row of the four finger images, with the numbers 1, 3, 5, and 7 displayed below them. Participants were asked to press the corresponding key on the computer keyboard to make their prediction. The experiment had a total of 72 explicit probe tests.

For participants in the Tone groups, each of the four fingers was additionally associated with pure tones with frequencies of 220, 440, 880, or 1,760 Hz (i.e., “A” note across four octaves). Tones were presented for the first 100 ms of each finger tap instruction—participants were instructed that tones would occur with the onset of each finger tap instruction, but were not informed that these would be redundant with the instructions. The mapping of finger (e.g., index finger) to tone was counterbalanced across participants. It is well established that differences in pitch (i.e., frequency) influence perceived loudness (Stevens, 1934; Robinson and Dadson, 1956; Molino, 1973); however, for these frequencies, differences in perceived loudness have been shown to be minimal (ISO-226, 2003; Glasberg and Moore, 2006). Mean loudness of the tones was measured at the approximate position of the participants’ head using a Dawe Sound Level Meter 1400G (Dawe Instruments Ltd., London, United Kingdom) and was between 60 and 70 dB for the 4 tones. Participants in the Silent groups experienced the same visual presentation but did not receive any auditory cues.

## Results

### Explicit Knowledge

Explicit knowledge of the statistical learning was measured as accuracy on the probe tests, where participants were asked to predict the next finger tap instruction. Predictions of the highest likelihood response were scored as correct, not necessarily based on the instruction that occurred next. As there were 72 explicit probe tests throughout the experiment, we divided the overall experimental task into quarters of four blocks each, rather than the 16 sequence blocks, such that there would be a sufficient number of trials in each unit of analysis. As such, there are 18 explicit probes per experiment quarter.

**FIGURE 2:**
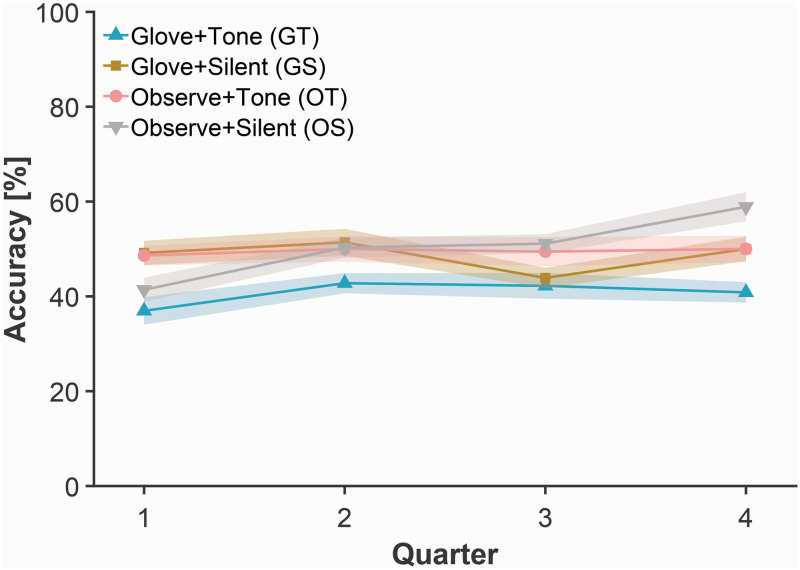
Mean accuracy in explicit probe tests for each group across quarters of the experimental task. Shaded bands represent standard error of the mean, adjusted for inter-individual differences.

**FIGURE 3:**
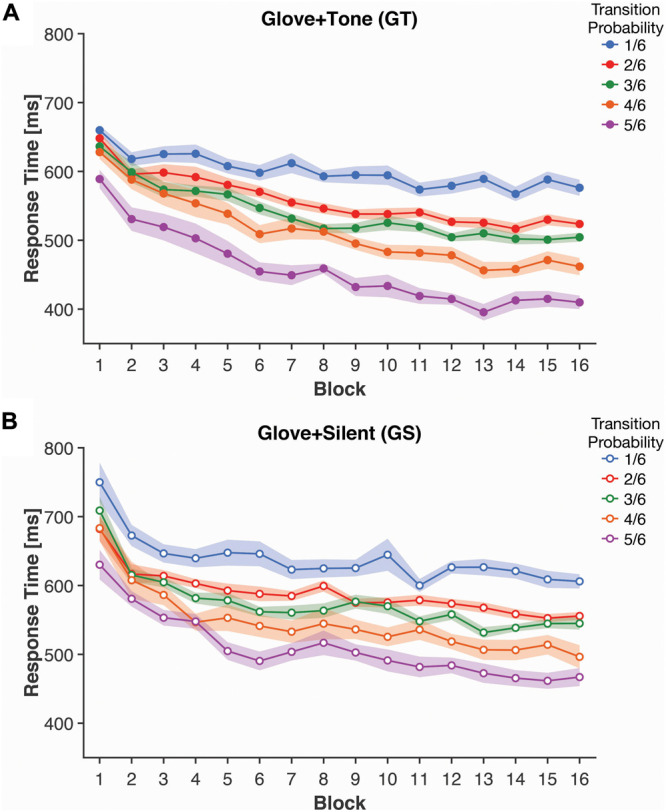
Mean response times for each transition probability and block, for both **(A)** Glove + Tone (GT) and **(B)** Glove + Silent (GS) groups. Shaded bands represent standard error of the mean, adjusted for inter-individual differences.

Accuracy on the explicit probe tests was analyzed using a 2 (Glove) × 2 (Tone) × 16 (Block) mixed ANOVA. As expected, accuracy on these probe trials improved across blocks [*F*(3, 76) = 7.13, *p* = 0.009, η*_p_*^2^ = 0.086] as shown in Figure 2, confirming that participants were acquiring explicit knowledge of the task, not procedural learning of the transition probabilities (discussed below; see Figure 3). The between-groups main effects and most interactions were non-significant; only the Block × Glove × Tone interaction was significant [*F*(1, 76) = 4.70, *p* = 0.033, η*_p_*^2^ = 0.058].

Here we conducted a 2 (Glove) × 2 (Tone) between-group ANOVA on the explicit probe accuracy from the first quarter of the task (i.e., blocks 1–4), following from a simple main effects approach. We observed a significant Glove × Tone interaction [*F*(1, 76) = 5.61, *p* = 0.020, η*_p_*^2^ = 0.066]. Results indicate that participants who had either the glove or the tones, but not both, performed best [mean (SEM) accuracy—GT: 36.9% (2.9); GS: 49.2% (2.6); OT: 48.6% (2.1); OS: 41.4% (2.6)]. A comparable 2 × 2 between-groups ANOVA was conducted on the last quarter of the task (i.e., blocks 13–16). Here we observed significant main effects of Glove [*F*(1, 76) = 5.49, *p* = 0.022, η*_p_^2^* = 0.067] and Tone [*F*(1, 76) = 5.49, *p* = 0.022, η*_p_^2^* = 0.067], but no significant interaction [*p* = 0.97].

We then examined the change between the first and last quarters using paired-samples *t*-tests for each group. Mean accuracy improved, but the improvement was nominal in magnitude for all groups except for the Observe + Silent group—where the improvement was more definite [mean (SEM) accuracy—GT: 40.8% (2.1); GS: 50.0% (2.6); OT: 50.0% (2.8); OS: 58.9% (3.1); three *p*’s > 0.05, except OS: *t*(19) = 3.43, *p* = 0.003, Cohen’s *d* = 1.03].

### Implicit Knowledge

Implicit knowledge of the statistical learning was measured as the response time (RT) for the finger tap responses; as such, this analysis only applies to participants that wore the computer glove (GT and GS groups). Here we calculated the mean response time for trials based on their Transition Probability [1, 2, 3, 4, or 5 (out of 6)]; response times are shown in Figure 3.

Response times was initially analyzed as a 2 [Tone: Tone (Group GT) vs. Silent (Group GS)] × 16 (Block) × 5 (Transition Probability) mixed ANOVA. Here we observed a significant effect of Block [*F*(15, 570) = 29.76, *p* < 0.001, η*_p_*^2^ = 0.439], with faster response times as participants progressed through the experiment. The main effect of Transition Probability was also significant [*F*(4, 152) = 51.12, *p* < 0.001, η*_p_*^2^ = 0.574], with faster responses for the higher probability transitions. The Block × Transition Probability interaction was also significant [*F*(60, 2,280) = 2.58, *p* < 0.001, η*_p_*^2^ = 0.064] and was investigated with further ANOVAs following from a simple main effects approach. However, we did not observe a significant main effect of Tone [*F*(1, 38) = 2.56, *p* = 0.12, η*_p_*^2^ = 0.063] nor were any interaction effects including tone statistically significant [all *p*’s > 0.1].

To further characterize the interaction, we conducted two additional ANOVAs. The first ANOVA averaged the response times across the first four blocks (i.e., blocks 1–4) and was examined as a 2 (Tone) × 5 (Transition Probability) mixed ANOVA. The factor of Tone was included as a planned factor as multisensory learning was the focus of the study. Response time was significantly faster for higher Transition Probability [*F*(2, 88) = 15.92, *p* < 0.001, η*_p_*^2^ = 0.295]. Response times were 98.0 ms faster for the highest probability transitions than the lowest probability transitions. This finding was expected and also suggests that RT reflects initial learning of the transition structure. However neither the main effect of Tone [*F*(1, 38) = 1.02, *p* = 0.32, η*_p_*^2^ = 0.026] nor the interaction [*F*(2, 88) = 0.41, *p* = 0.69, η*_p_^2^* = 0.011] was significant—indicating that addition of a tone was not faciliatory initially.

A similar 2 × 5 mixed ANOVA was conducted based on the last four blocks (i.e., blocks 13–16). The effect of Transition Probability on response times persisted [*F*(2, 88) = 72.48, *p* < 0.001, η*_p_*^2^ = 0.656] and increased in magnitude relative to the first blocks [*F*(3, 108) = 12.05, *p* < 0.001, η*_p_*^2^ = 0.241] (directly compared through a *post hoc* analysis), demonstrating continued learning of the task structure. Response times were 160.5 ms faster for the highest than the lowest probability transitions. The interaction with tone remained non-significant [*F*(2, 88) = 0.51, *p* = 0.63, η*_p_*^2^ = 0.013], however, the main effect of tone was significant in the last four blocks [*F*(1, 38) = 4.56, *p* = 0.039, η*_p_*^2^ = 0.107], where participants who received the auditory cues had a mean response time that was 41.6 ms faster—potentially indicating a facilitatory effect of the additional cues. Admittedly, this effect is relatively weak and was examined as a planned comparison, rather than in follow-up to an interaction involving tone from the full ANOVA that included all blocks.

## Discussion

The purpose of this study was to examine the relationship between auditory and visual input on implicit and explicit measures of a statistical motor-learning paradigm as it applies to hand motor commands. The primary question of multi-modality vs. modality specificity in statistical learning has been debated in the literature with some research strongly supporting the idea of multiple neural systems (Conway and Christiansen, 2005) and other research favoring the existence of a domain-general learning system (Kirkham et al., 2002). Our study uniquely contrasted a motor-learning paradigm with an observation paradigm for finger-movement sequences that were presented visually, with and without accompanying auditory tones that paired a specific pitch with a particular finger movement. The results showed two main findings, one related to explicit measures of statistical motor-learning and one related to implicit measures. Each will be discussed in turn, followed by a general discussion of our results within the larger framework of modality specificity vs. domain generality in statistical learning.

Our first result was that when auditory tones were paired with the visual movement cues, explicit probe performance decreased in the tapping task (i.e., Group GS > GT) but increased in the observation task (i.e., Group OT > OS). That is, the tones appeared to distract overt motor performance, but enhance performance associated with passive observation. Interestingly, this was only observed in early blocks of the task, and was attenuated in later blocks. One possible explanation for these findings is that in the tone groups, the task consisted of three input modalities for action (visual, auditory, movement), but only two were present for observation (visual, auditory). It is well known that auditory stimuli can be arousing (Eason et al., 1969; Paus et al., 1997), with both enhancing (Driver and Spence, 1998) and distracting effects (Escera et al., 2000). Thus, in the early blocks of our study, the movement (Glove + Tone; GT) group may have been optimally supported by a relatively simple visual-to-motor representation that was not available in the Observe + Tone (OT) group. This may have been related to diminished performance due to an information overload not essentially required (i.e., redundant) for the tapping task. On the other hand, the Observe + Tone group lacked the kinesthetic input afforded in the Glove + Tone group, and here the addition of more sensory input may have aided observation performance. While it has been shown that action observation can support motor learning (Mattar and Gribble, 2005), in our study, the observation groups (OT and OS) did not contain movement observation, but rather an observed *static* visual representation of the subsequent finger transition to be learned. Thus a combination of multisensory (visual-auditory) cues may have benefited the declarative task in the observation groups but not in the movement groups where the auditory information was redundant (Kalyuga et al., 1999). Interestingly, these effects disappeared in the last four blocks of the task suggesting that at some point in the skill-learning process (Fitts, 1964), the respective distracting and beneficial effects were no longer implicated in performance. Indeed, probe accuracy improved across blocks with the largest improvements occurring in the Glove + Tone (3.9%) and Observe + Silent (17.5%) groups. Thus, with repeated trials, the group with the least information input (Observe + Silent) became more like all the other groups that were richer in encoding input.

The second main finding was that the glove response times improved with successive trial blocks such that the last four blocks were faster than the first four blocks (Figure 3). However, in the last four blocks, the presence of auditory tones decreased response times in the finger-tapping task compared to the silent group. Thus the presence of tones enhanced the implicit measure of motor learning, perhaps reflecting the progression of knowledge of the task from a more conscious representation in the earlier blocks to something more automatic in later blocks (Masters, 1992). This idea is consistent with the explicit Glove + Tone group results discussed above where the distracting effects of the tones on the probe task disappeared in later trials compared to earlier ones. It has been shown that the implicit motor learning of a sequence can be improved 12 h following an intervening explicit memory task. The authors argue that this effect is due to off-line procedural improvements subserved by a fundamental dissociation between explicit-declarative and implicit-procedural memory systems, and neuroplasticity (Brown and Robertson, 2007). Presumably the hippocampus is a key player in the declarative memory circuit (Eichenbaum, 2000), whereas motor learning of a sequence involves many pre-motor and motor areas that decrease in their activity as learning progresses (Toni et al., 1998). Furthermore, Schendan et al. (2003) showed that both explicit and implicit measures of SRTT was associated with hippocampal/medial-temporal-lobe activation suggesting that procedural learning is also subserved by a memory system that *overlaps* with declarative memory. Such an overlap might be the reason that we found a link between the effects of the auditory tones on both the explicit and implicit measures in the motor-learning task. It is additionally worth considering, however, that the synchronous auditory and visual cues may have increased the saliency of the visual cues, following from the well-known pip-and-pop effect (Van der Burg et al., 2008). This could be evaluated in an additional group where the visual and auditory cues are both still presented, but with an offset in their presentation. If the response time improvements related to the auditory tones are attenuated, this would provide further specificity in how the multisensory cues were combined, such that the synchronicity of presentation was relevant—and potentially shares the same attentional processing as the pip-and-pop effect.

While we describe the non-glove groups as “Observe” as this is what we can assess behaviorally—that is, they did not make any overt motor movements, the instructions were more precisely asking them to engage in movement imagery (i.e., “Imagine” as a group descriptor). Several studies have shown that motor/movement imagery involves engaging similar networks of brain regions as actual movements (Lotze et al., 2003; Lotze and Cohen, 2006; Madan and Singhal, 2012; Hardwick et al., 2018; Kline et al., 2020). The intention here was to minimize actual movements as the experimental manipulation, but still encourage active engagement in the presented finger-tapping sequences through movement imagery and not allowing participants’ minds to wander. As the participants in the Observe groups performed comparably to the Glove groups in the explicit knowledge test, and on average were numerically better, it does seem that the Observe participants were engaged in the task and did learn sequences.

Taken together, here we found that participants that only had to observe visual cues performed best in the explicit probe tests—with both enactment and additional auditory tones distracting from explicit knowledge. In contrast, when evaluating implicit knowledge (as measured by glove RT), we found that the additional auditory cues were *beneficial* to performance. These findings indicate that explicit knowledge of the statistical learning was best with only the single modality, but implicit knowledge was best when all three modalities were involved. Moreover, the results demonstrated a clear main effect of transition probability on response time and hastening of response time as learning accumulated (Figure 3).

Regarding the domain generality vs. modality-specificity of statistical learning, our results support the latter in the case of a motor-learning paradigm. Frost et al. (2015) has proposed a theoretical model to account for sensory modality effects on statistical learning with both separate and shared systems, particularly between auditory and visual inputs. A key concept in this model is that a brain region like the medial temporal lobe could support computational generality in statistical learning as it does for memory (Bogaerts et al., 2016), but this circuit will still have unique connections with individual sensory cortices that support modality-specific representations (Frost et al., 2015). On the basis of our findings, we argue in favor of this theoretical approach, and suggest that it can easily accommodate our observations in the motor-learning domain for hand motor commands that demonstrate both a convergence and a dissociation of modality effects on explicit and implicit measures of statistical learning.

The computer glove procedure used here generally worked well, while also being sufficiently different than more established procedures used in AGL and SRTT studies. However, future work may want to include a computer keyboard or response pad group as a comparison to the computer glove and also include a random-sequence control group to allow for distinct estimates of practice and learning effects. These findings add to the growing literature demonstrating different mechanisms underlying explicit and implicit knowledge, convergent with the notion of multiple distinct systems supporting motor skill learning (e.g., Clark and Ivry, 2010).

## Data Availability Statement

The raw data supporting the conclusions of this article will be made available by the authors, without undue reservation, to any qualified researcher.

## Ethics Statement

The studies involving human participants were reviewed and approved by the University of Alberta Institutional Review Board. The patients/participants provided their written informed consent to participate in this study.

## Author Contributions

CM implemented the experiment, oversaw data collection, analyzed the data, and wrote the first draft of the manuscript. Both authors conceptualized the study, edited the manuscript, and approved the final draft.

## Conflict of Interest

The authors declare that the research was conducted in the absence of any commercial or financial relationships that could be construed as a potential conflict of interest.
